# Parental views on plastic surgery for Down syndrome: an African perspective

**DOI:** 10.11604/pamj.2019.32.207.18316

**Published:** 2019-04-29

**Authors:** Afieharo Igbibia Michael, Olumide Olatokunbo Jarrett

**Affiliations:** 1 Department of Plastic, Reconstructive and Aesthetic Surgery, University, College Hospital, Ibadan, Oyo State, Nigeria; 2 Department of Paediatrics, Faculty of Clinical Sciences, College of Medicine, University of Ibadan, Nigeria

**Keywords:** Parents, down syndrome, plastic surgery, ethics, prominent tongue, mothers, Africa, attitudes, awareness, stigmatization

## Abstract

**Introduction:**

Plastic surgery for Down syndrome has not been embraced in sub-Saharan Africa. This study sought to determine the attitudes of some parents from sub-Saharan Africa to plastic surgery for their Down syndrome child.

**Methods:**

Consenting parents completed a questionnaire survey instrument that obtained demographic characteristics and contained a likert scale on attitudes to plastic surgery. Internal consistency of the scale was determined with Chronbach's alpha and Pearsons chi square analysis was used to analyze relationships between demographic variables and attitudes scores. Values less than 0.05 were considered statistically significant.

**Results:**

Most (61.9%) of the 42 consenting mothers were above 35 years of age. The most disturbing of the Down syndrome characteristics were the protruding tongue, 18(42.9%), slanting palpebral fissures, 14(33.3%) and the flattened nasal bridge 14(33.3%). Although the mothers had low awareness of plastic surgery most of them had favourable attitudes towards it. A reliability analysis of the mother's attitudes on the likert scale showed good internal consistency. Chronbachs alpha 0.87.

**Conclusion:**

The parents in this study have favourable attitudes towards plastic surgery for Down syndrome. The prominent tongue was the most disturbing feature.

## Introduction

The Down syndrome child has characteristic facial stigmatizing features. These include a large tongue, flattened nasal bridge, small chin, the presence of epicanthal folds and a broad neck [1, 2]. These aesthetic shortcomings can also be functionally limiting for example the large tongue could result in breathing difficulties, drooling of saliva from the mouth and speech limitations [2]. Plastic surgery procedures done to erase these features are believed to improve societal acceptance of the child [3, 4]. This has however been met with some criticism. It has been proffered that energies should be harnessed towards improving societal attitudes to these patients rather than subjecting the patients to painful procedures [5-7]. While some parents consider plastic surgery for Down syndrome as cruel, others feel that the improvement in appearance following plastic surgery would lead to a reduction in societal prejudices and improved societal functioning [3-5]. Down syndrome though not rare in sub Saharan Africa, is largely understudied and underreported [8, 9]. This study sought to obtain parental views towards plastic surgery for Down’s syndrome. The findings from this study would be instructive for counseling sessions for these procedures.

## Methods

This was a cross sectional survey of parents of children with Down syndrome who presented at the paediatric genetic outpatient clinic of the University College Hospital, Ibadan between October 2016 and October 2017. An Informed consent was obtained from all participating parents and a structured 27-item questionnaire was administered. Demographic data obtained included the age of the child, age of the parents at the birth of the child with Down syndrome, birth order of the child and the family setting. Awareness of plastic surgery for Down syndrome and body region of concern were sought. A likert scale assessing the attitude of the parents to plastic surgery for their wards had five levels of agreement from strongly agree to strongly disagree. Descriptive statistics were used to analyze the demographic variables and levels of agreement on the likert scale. Internal consistency of the scale was determined with Chronbach's alpha and Pearsons chi square analysis was used to analyze relationships between demographic variables and attitudes scores. Values less than 0.05 were considered statistically significant.

## Results

**Figure 1:**
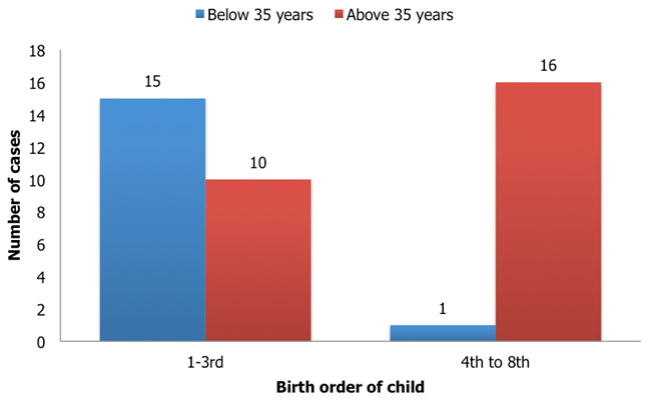
Relationship between age of mother and birth order of Down syndrome child (p < 0.05)

**Socio-demographic characteristics of the respondents:** Forty-two parents participated in the study. All participating parents were mothers. The age range of the children was from 2 months to 8 years. Most of the children were males. Twenty-six (61.9%) of the mothers were above 35 year of age at the birth of the child while only 17 (40.5%) of the fathers were above 45 years of age at the birth of the child. More than half of the children were of the birth order three and below Table 1. We found that Down syndrome occurring in children of birth order of four and above were more likely to be borne to mothers who were older than 35 years of age. This was statistically significant (p < 0.05) Figure 1.

**Table 1:** Socio-demographic characteristics of the respondents

Variable	Number	Percent
	N=42	
**Age of Child (months)**		
1-6	8	19
6-12	11	26.2
13-18	6	14.3
19-24	8	19.0
>24	9	21.4
**Gender of children**		
Female	11	26.2
Male	31	73.8
**Age of Mother at birth of the child**		
<35years	23	54.8
>35years	17	40.5
Missing value	2	4.8
**Age of Father at birth of child**		
<45years	23	54.8
>45years	17	40.5
Missing values	2	4.8
**Family setting**		
Monogamous	35	83.3
Polygamous	6	14.3
Divorced	0	
Separated	0	
Never married	1	2.4
**Birth order of child**		
1^st^	6	14.3
2^nd^	7	16.7
3^rd^	10	23.8
4^th^	4	9.5
5^th^	12	28.6
6^th^	0	0.0
7^th^	0	0.0
8^th^	1	2.4
Only child	2	4.8

**Figure 2:**
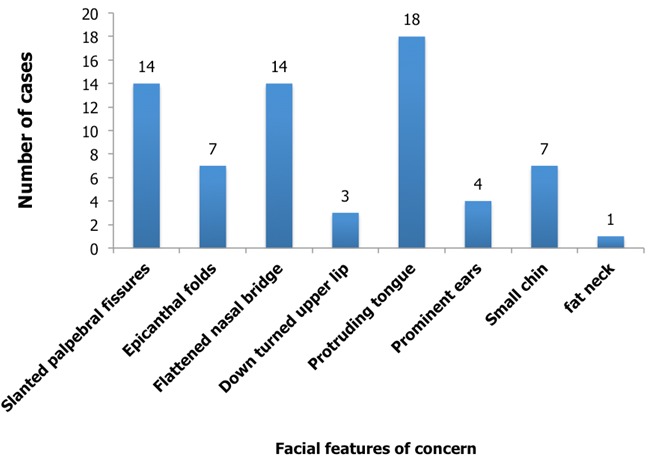
Facial features of concern to mothers of children with Down syndrome

**Facial characteristics of concern to the mothers:** Figure 2 shows the more common Down syndrome facies of concern to them were the protruding tongue, 18 (42.9%), slanting palpebral fissures, 14 (33.3%) and the flattened nasal bridge 14 (33.3%).

**Figure 3:**
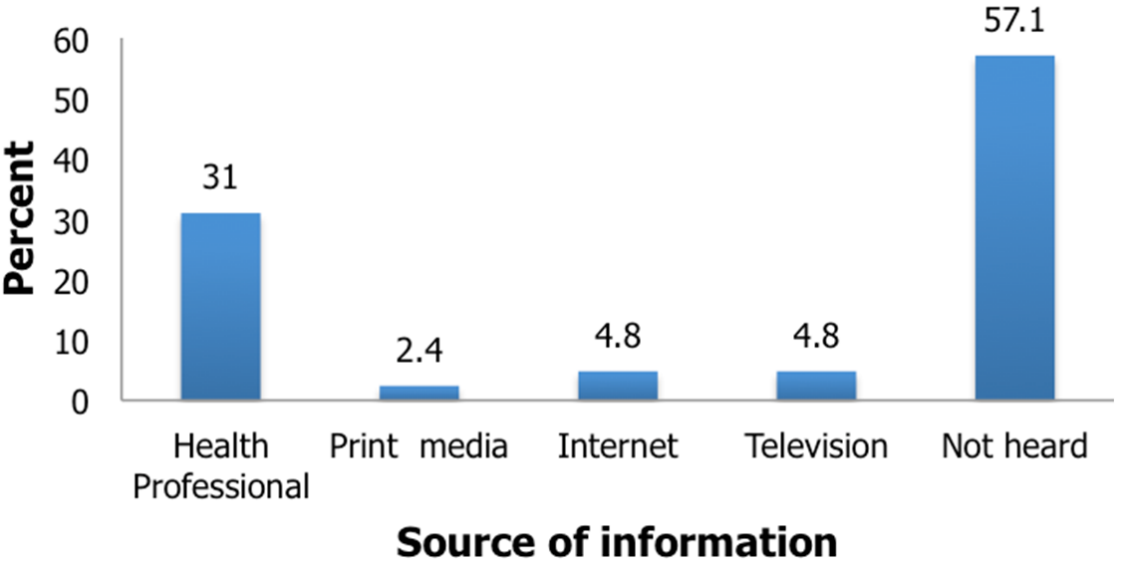
Source of awareness of mothers to plastic surgery for Down syndrome

**Awareness of the mothers on plastic surgery:** Most of the mothers were not aware that plastic surgery could be done for the Down syndrome child. For those who were aware, the main source of information was from a health professional, Figure 3. Despite this low awareness on plastic surgery, most, 34(81%) of the mothers felt it was right to request plastic surgery for a Down syndrome child.

**Figure 4:**
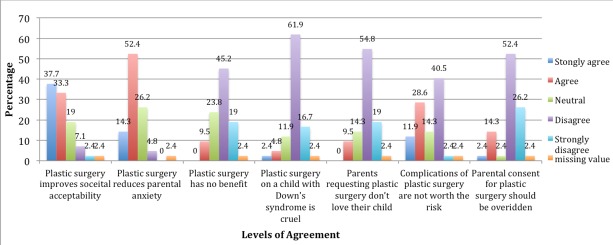
Overview of level of agreement of the mothers to Likert scale questions

**Level of agreement to likert scale questions of plastic surgery for Down syndrome:** There was a high level of agreement by the mothers that plastic surgery improves societal acceptability and parental anxiety. The mothers did not believe that plastic surgery was cruel or suggested a lack of love of the parent for the child. Figure 4 shows an overview of these findings.

**Reliability of the likert scale:** A reliability analysis of the mother's attitudes on the likert scale showed good internal consistency. Chronbachs alpha was 0.87. All items resulted in a decrease in the alpha if deleted; as such none of the questions were required to be deleted.

**Attitude to plastic surgery:** The mean attitude score was 33. Twenty-five (59.5%) mothers had scores above the mean and therefore positive attitudes to plastic surgery while 16 (38.1%) had scores below the mean. Neither the age of the mothers, gender of the child or awareness of plastic surgery were significantly associated with their attitudes Table 2.

**Table 2:** Pearson’s chi square analysis of mother’s attitude to plastic surgery

Variable	Attitude		P-value
	Positive	Negative	
**Mothers age**			
<35 years	8	8	0.25
>35 years	17	8	
**Gender of child**			
Male	2	6	0.11
Female	21	10	
**Awareness of plastic surgery**			
Yes	10	8	0.53
No	15	8	

## Discussion

Down syndrome is not uncommon in sub-Saharan Africa [8, 9]. Though literature on Down syndrome from Sub-Saharan Africa is sparse, an incidence of 1 in 865 live births has been reported [8]. It has been established that the risk of having a child with Down syndrome increases with maternal age [8-10]. We found more of the mothers in this study to be over 35 years of age at the birth of the Down syndrome child. Adeyokunu reported an increased incidence of younger mothers with Down syndrome with a mean age of 28.06 years [8]. From our study later children were more likely to have Down syndrome if the mothers were older. The sex ratio of 2.8 in this study is much higher than has been quoted in other studies [8, 10, 11] and while Down syndrome is seen in children of birth order above four [8, 9] we found that for majority of children, the birth order was 3 and below. The six characteristic facial features in a child with Down syndrome are the presence of epicanthal folds, oblique palpebral fissures, flattened nasal bridge, a large protruding tongue (macroglossia), hypotonic lower lip and macrogenia (receding chin) [1, 2]. These are present to varying degrees in these children. The mothers in our study found the face to be of most concern than other parts of the body. The most prominent of the facial features being the protruding tongue while others were the slanting palpebral fissures and the flattened nasal bridge. The large tongue could be functionally limiting. It can be associated with breathing difficulties, persistent drooling of saliva and impaired speech [1]. Partial glossectomies have proved helpful in relieving these symptoms that accompany the macroglossia. In Olbrichs [1] review of 102 patients who had had plastic surgery, over 80% of the parents reported a reduction in mouth breathing, improved speech, drooling and eating following the correction of the protruding tongue. There was also improved social functioning of these children. He recommended that plastic surgery for Down syndrome be done from age 3, as this would improve the quality of life of the child and the parents. Facial plastic surgery for Down syndrome done to correct or blur these the facial features also results in less stigmatization, improved parental, societal and patient functioning and therefore improved quality of life [1-4, 12].

The ethical justification for these surgeries have however been largely questioned [5-7]. Studies have shown that there was no significant improvement in patient functioning or societal acceptance [13-15]. There have been arguments that more efforts should be made towards improving societal acceptance of these children than changing the child to suit the society [5-7, 16]. Jones considers these procedures very unnecessary and strongly recommends that they should be outlawed [6]. The mothers in our study had a low awareness of plastic surgery procedures for Down syndrome, despite this most of them had very positive attitudes towards it. Their desire for plastic surgery was mostly to increase societal acceptance of the child. Two main assumptions can be made from this. The first is the low level of awareness suggests that they also are not aware of the possible risks and complications of the procedure. Secondly the need for increased societal acceptance may be a reflection of the still stigmatizing nature of their sociocultural terrain. In Africa stigmatization of people living with disabilities is high resulting in the parents shielding their child from the society or out rightly rejecting the child [17, 18]. In Goeke's much larger study of parental opinions towards plastic surgery, most of the parents were averse to the procedure [5]. The parents sampled which represented approximately 50% of the actual sample, were members of an advocacy group for Down syndrome and were well adjusted to the status of their children [5]. The majority of the parents in another study were also concerned about the risks of the procedure to the child [15]. Parental awareness and society therefore play major roles in decision making for facial plastic surgery in Down syndrome [5, 16].

## Conclusion

The parents in this study have positive attitudes towards plastic surgery for their children living with Down syndrome. The most prominent of these features is the protruding tongue. Adequate counseling would be required in offering facial plastic surgery. There may be need to investigate more into the societal challenges faced by parents of children with Down syndrome.

### What is known about this topic

- The facial characteristics of Down syndrome are stigmatizing;
- Children with Down syndrome face societal isolation.

### What this study adds

- Parents of children with Down syndrome have positive attitudes to plastic surgery for these children;
- The protruding tongue is the most disturbing facial feature.
